# Numerical Simulation of Rubber Concrete Considering Fatigue Damage Accumulation of Cohesive Zone Model

**DOI:** 10.3390/ma17205018

**Published:** 2024-10-14

**Authors:** Cai Liu, Houmin Li, Kai Min, Wenchao Li, Keyang Wu

**Affiliations:** 1 School of Engineering, Architecture and the Environment, Hubei University of Technology, Wuhan 430068, China; liucai1357@163.com (C.L.); 102200768@hbut.edu.cn (K.M.); 18954340691@163.com (W.L.); 2 Wuhan Construction Engineering Co., Ltd., Wuhan 430056, China; wukeyang@wceg.com.cn

**Keywords:** rubber concrete, cohesive zone model, mesoscale modeling, interfacial transition zone, fatigue life

## Abstract

Rubber concrete (RC) has been used in fatigue-resistant components due to its durability, yet the numerical simulation of its fatigue properties remains in its early stages. This study proposes a cohesive zone model (CZM) that accounts for the accumulation of fatigue damage at the mesoscale to investigate the fatigue performance of RC. The model integrates static and fatigue damage in the CZM, effectively capturing damage caused by fatigue loading. Validation was conducted using experimental data from the existing literature. Based on this validation, four concrete beams with varying rubber replacement rates (0%, 5%, 10%, and 15%) were tested. The CZM was employed to describe the mechanical behavior of the interface transition zones (ITZs) and the mortar interior, which were simulated and analyzed under different stress levels. The results demonstrate that the model accurately simulates crack propagation paths, interface damage evolution, and the fatigue life of RC beams under fatigue loading. A functional relationship between fatigue life and stress level was established for various rubber replacement rates. This study provides a reference model for numerical simulations of RC under fatigue loading conditions and introduces new approaches for analyzing the fatigue performance of other materials.

## 1. Introduction

In recent years, the automotive industry has grown significantly, leading to a dramatic increase in the use of automobile tires and the production of many waste tires. Consequently, recycling waste tires has emerged as a global concern. Waste tires represent a significant source of waste rubber, a polymer material noted for its excellent elasticity [1]. Relevant studies have indicated that rubber particles randomly dispersed in cement can absorb micro-expansion and contraction, thereby enhancing the brittle resistance of concrete [2,3]. RC combines the properties of both rubber and concrete, characterized by a low modulus of elasticity, superior deformation resistance, excellent crack resistance, and significant energy absorption capacity [4]. Gerges et al. [5] conducted experiments measuring the compression, tensile, and impact loads on concrete cylinders with varying rubber admixtures, finding that RC exhibits lower density, enhanced toughness, and increased impact resistance compared to ordinary concrete. Furthermore, Liu et al. [6] demonstrated through real-time monitoring using the acoustic emission technique that RC offers improved fatigue life and fracture energy over ordinary concrete.

With the continuous advancement of computer performance, concrete study is no longer confined to experimental research. Scholars have started to employ numerical simulation methods and investigate the mechanical properties of concrete from different scales. Based on the scale of the study, current numerical simulations of concrete structures can be broadly classified into microscale, mesoscale, and macroscale [4]. Among them, the microscale is investigated at the atomic and molecular level, which is not widely applicable due to the small scale of its study, making it challenging to explore the effect of molecular composition on the overall mechanical properties using the available means at this stage. At the mesoscale, concrete is primarily considered a multiphase heterogeneous material [7], and numerical computations of the structure can be achieved by defining each material phase’s properties and mechanical behaviors. Currently, most scholars also prefer to study the mechanical properties of concrete at this scale [8,9,10]. At the macroscale, the concrete material is equivalent to an isotropic material, neglecting the complex internal structure of concrete, which is only applicable to overall calculations.

In previous studies, the mesoscale modeling of concrete has focused on developing multiphase models representing aggregates, mortar, and interfacial transition zones. When subjected to loading, these phases interact, influencing the overall mechanical properties of the concrete member. Currently, two principal methods exist for constructing mesoscale aggregate models of concrete. The first method employs digital imaging technology or CT scanning to capture the actual shapes, quantities, and locations of the aggregates in concrete, which are then transformed into a geometric model. Nitka et al. [11] developed a realistic aggregate model by utilizing X-ray technology to scan concrete and successfully simulated the bending damage of the material using a discrete element model. The second method involves writing a computer program to generate random aggregate models for each concrete component. Ouyang et al. [12] developed a novel stochastic aggregate placement method using a grid-based approach and validated the model by simulating the uniaxial compression of concrete. Although the first method produces the most realistic aggregate model, it is costlier and more complex during the finite element preprocessing stage. In contrast, the second method enhances modeling efficiency and meets the precision required for subsequent calculations, provided the aggregates are reasonably placed.

As research on concrete continues to advance, the mechanical interactions between its components should not be overlooked. Unlike the traditional continuum mechanical simulation, considering different material components brings problems such as discontinuous geometric features and stress singularities for numerical calculations. Therefore, scholars have adopted improved continuum mechanical simulation methods or new novel mechanical simulation methods to realize the study of concrete mesoscale for simulation studies, using, for example, plastic damage models [13,14], fracture phase field models [15,16,17], peridynamic models [18,19], and cohesive zone models [20,21]. The fracture phase-field model integrates concepts from phase-field theory and fracture mechanics to effectively simulate the complex behaviors of materials undergoing fracture. This model’s accuracy, however, is influenced by the width of the phase-field features, which can impact its performance. Moreover, additional research is necessary to characterize the ITZ in fine-scale models more accurately. The peridynamic model, a recent innovation in representing mechanical behaviors, utilizes a bond-based approach between particles. It is formulated using integral calculations, which inherently circumvent issues like material discontinuities’ geometrical features and stress singularities. However, it requires converting some commonly used material parameters before application. The CZM offers a numerical simulation method to describe a material’s fracture behavior. It simulates the material’s fracturing under load by introducing cohesive forces or fracture criteria. By embedding cohesive zone elements along the prospective crack path, this model allows the crack to extend spontaneously, driven by the energy dissipated during crack formation, eliminating the need for prefabricated cracks.

This study aims to develop a fatigue damage model applicable to RC. The model combines static and fatigue damage within a CZM, characterizing the mechanical behavior between components of RC under fatigue loading at the mesoscale. The model was validated against experimental results from the existing literature. Subsequently, four concrete beams with varying rubber substitution rates (0%, 5%, 10%, and 15%) were simulated to analyze crack propagation during fatigue loading, interface damage evolution, and fatigue life. A functional relationship between fatigue life and stress level was also established for different rubber replacement rates. This study provides a model reference for the numerical simulation of RC under fatigue loading.

## 2. Model Establishment

### 2.1. Constitutive Laws of CZM

The investigation in this paper focuses on implementing a CZM that considers fatigue damage accumulation. It builds upon a bilinear cohesive zone model (BCZM) designed for mixed-mode loading. Consequently, this section will elaborate on the classical theory of this model and the corresponding enhancements.

#### 2.1.1. Concrete Crack Expansion Forms

**Figure 1:**
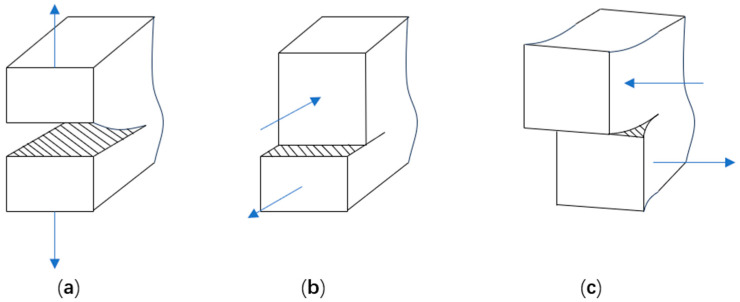
Three patterns of crack expansion:(**a**) opening mode, (**b**) sliding mode, (**c**) teaning mode.

Concrete crack extension patterns can be classified into three categories, namely Type I (opening mode), Type II (sliding mode), and Type III (tearing mode) [22], as illustrated in Figure 1. The damage in concrete under tension and compression is primarily governed by Type I fracture and composite fracture, respectively. Concrete’s inherent nonhomogeneity and multiphase nature can also influence the fracture model. Therefore, the model proposed in this paper integrates the effects of both Type I and composite cracks.

#### 2.1.2. Constitutive Laws for CZM under Monotonic Loading

**Figure 2:**
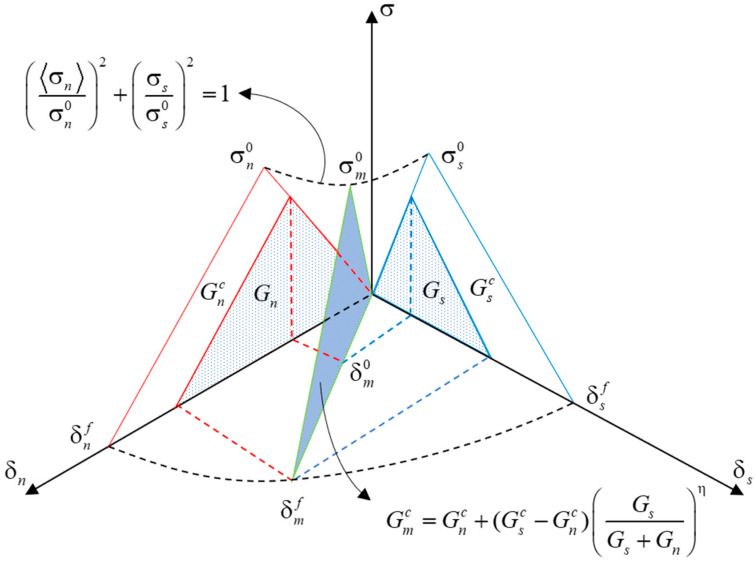
BCZM constitutive laws in mixed mode.

Given the complexity of internal stresses in concrete subjected to external forces, evaluating the variations in normal and shear traction forces within the cohesive zone model (CZM) is essential. Therefore, we employed the bilinear cohesive zone model (BCZM) in mixed mode, as proposed by Camanho [23], to characterize the damage behavior of concrete under static forces. The model incorporates normal and shear displacements along the crack by introducing a mixed-mode displacement $\delta_{m}$. It defines the damage initiation displacement $\delta_{m}^{0}$ and the final failure displacement $\delta_{m}^{f}$ in the mixed mode. These displacements are compared with the magnitude of $\delta_{m}$ to assess the degree of material damage under complex stress conditions. The BCZM constitutive laws in the mixed model are illustrated in Figure 2.

As shown in Figure 2, we adopted the squared stress criterion to determine the damage onset displacement $\delta_{m}^{0}$ in mixed-mode conditions, where damage onset occurs when the sum of the stress ratios in both directions equals one.

(1) $${\left(\frac{\langle \sigma_{n}\rangle }{\sigma_{n}^{0}}\right)}^{2}+{\left(\frac{\sigma_{s}}{\sigma_{s}^{0}}\right)}^{2}=1$$

where $\sigma_{n}$ and $\sigma_{s}$ denote normal and shear stresses, respectively. $\sigma_{n}^{0}$ and $\sigma_{s}^{0}$ represent the interface strength in normal and shear directions. <> is the Macaulay symbol, which has a value of 0 in the compressed state, indicating that simple compression does not induce damage. The mixed-mode displacement $\delta_{m}$ can be expressed as follows:

(2) $$\delta_{m}=\sqrt{{\langle \delta_{n}\rangle }^{2}+\delta_{s}^{2}}$$

where $\delta_{n}$ and $\delta_{s}$ denote normal displacement and shear displacement, respectively.

The material enters a damage evolution state when the mixed-mode displacement $\delta_{m}$ surpasses the damage initiation threshold $\delta_{m}^{0}$ but remains below the final failure displacement $\delta_{m}^{f}$. This stage is elaborated in the paper through the application of the Benzeggagh–Kenane energy criterion [24], described as follows:

(3) $$G_{m}^{c}=G_{n}^{c}+(G_{s}^{c}-G_{n}^{c}){\left(\frac{G_{s}}{G_{s}+G_{n}}\right)}^{\eta }$$

where $G_{m}^{c},G_{n}^{c}$, and $G_{s}^{c}$ denote the mixed fracture energy, normal fracture energy, and shear fracture energy, respectively. η represents the mixed-mode fracture energy coefficient. During the damage evolution stage, the monotonic damage variable $D_{m}$ is introduced to describe the cohesive element’s progression from the damage initiation state to the final damage state. Its mathematical expression is as follows:

(4) $$D_{m}=\frac{\delta_{m}^{f}(\delta_{m}^{\max}-\delta_{m}^{0})}{\delta_{m}^{\max}(\delta_{m}^{f}-\delta_{m}^{0})}$$

where $\delta_{m}^{max}$ indicates the maximum mixed-mode displacement in the whole loading course. When $D_{m}=0$, it signifies no damage, with the material interface remaining intact. $D_{m}=1$ indicates complete material damage, resulting in cracks in the interface. For values of $D_{m}$ between 0 and 1, the interface undergoes a damage evolution stage during which both stiffness and traction force decrease. The incremental form of the monotonic damage variable in this evolution stage is as follows:

(5) $${\dot{D}}_{m}=\frac{\delta_{m}^{0}\delta_{m}^{f}{\dot{\delta }}_{m}}{\left(\delta_{m}^{f}-\delta_{m}^{0}\right)}\frac{1}{\delta_{m}^{2}}$$

#### 2.1.3. Constitutive Laws for CZM under Fatigue Loading

**Figure 3:**
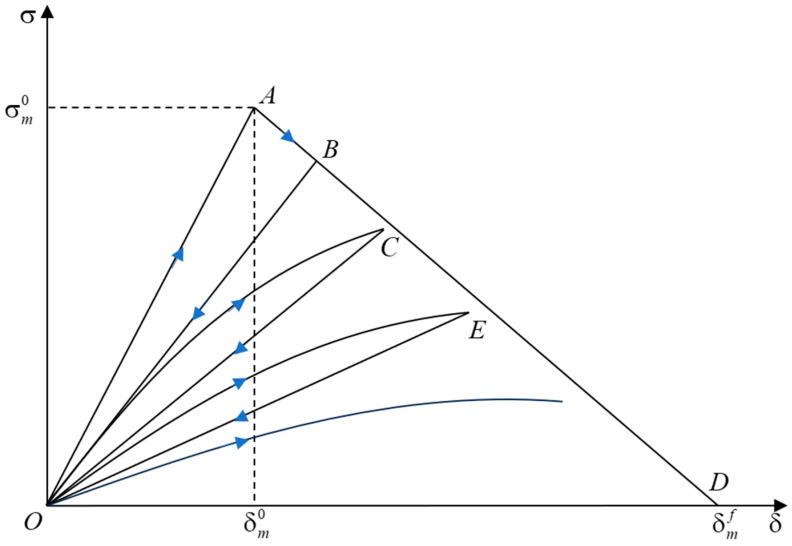
Constitutive laws for CZM under fatigue loading.

This study integrates the law of fatigue damage accumulation based on the CZM applicable to monotonic loading damage in Section 2.1.2. This integration enables the model to accumulate static load damage as well as apply to fatigue. The CZM constitutive law for fatigue loading is illustrated in Figure 3.

The constitutive laws for the CZM under fatigue loading divide fatigue loading into three stages: loading–unloading–reloading. The OA stage is the linear elasticity stage under monotonic loading conditions, in which the stiffness does not change, and damage does not occur. When the mixing displacement $\delta_{m}$ is greater than the damage onset displacement $\delta_{m}^{0}$ into the softening stage under monotonic static loading (AB stage), the material’s irreversible damage stiffness decreases; $D_{m}$ is the current moment of the damage accumulation, calculated by Equation (4), and at this time, the interfacial traction force decreases with an increase in the mixing displacement. The BO stage corresponds to the unloading phase, where no damage accumulation occurs. During subsequent loading in the $\hat{OC}$ segment, the presence of fatigue damage alters the response; instead of following the initial unloading slope, the traction force now follows a curved trajectory up to point C. Throughout this phase, the interface strength decreases gradually. The interface strength remains stable during the subsequent unloading process in the CO segment. Each cycle of fatigue loading results in progressive damage accumulation and a continuous reduction in interface strength, culminating in material failure.

This study introduces the total damage variable $D_{t}$ to differentiate the damage accumulation processes attributable to varied loading conditions. This variable represents the cumulative effect of monotonic softening and fatigue damage during the initial loading phase, whereas only fatigue damage is considered in subsequent loading phases, excluding the effects of monotonic softening. Upon establishing $D_{t}$, it is possible to derive the current normal and shear strengths of the interface, denoted as $\sigma_{n}$ and $\sigma_{s}$, respectively, from their initial values $\sigma_{n}^{0}$ and $\sigma_{s}^{0}$:

(6) $$\sigma_{n}=\sigma_{n}^{0}(1-D_{t}),\sigma_{s}=\sigma_{s}^{0}(1-D_{t})$$

The fatigue damage evolution equation must effectively represent the damaged state of cohesive elements under fatigue loading. In this study, we utilized the theory proposed by Roe [25] to describe the fatigue damage:

(7) $${\dot{D}}_{c}=\frac{\left|\Delta \dot{\bar{\delta }}\right|}{\delta_{\Sigma }}\left[\frac{\bar{T}}{\sigma_{m}^{0}(1-D_{t})}-\frac{\sigma_{f}}{\sigma_{m}^{0}}\right]H(\Delta \bar{\delta }-\delta_{m}^{0}),{\dot{D}}_{c}\geq 0$$

where the symbol $\Delta \bar{\delta }$ denotes the effective mixed displacement, which is equal to the displacement in the mixed mode of Equation (2) in the above equation. $\delta_{\Sigma }$ denotes the cumulative cohesion length, which determines the cumulative effective displacement required to destroy the cohesive zone. *H*(*x*) denotes the Heaviside step function, which is used to control whether or not fatigue damage is cumulative. $C_{f}$ is the control threshold for fatigue limit, which is taken as 0.1 in this paper. $\Delta \dot{\bar{\delta }}$ denotes the incremental form of the effective mixing displacements, which is defined in the subroutine as follows:

(8) $$\Delta \dot{\bar{\delta }}=\Delta {\bar{\delta }}_{t}-\Delta {\bar{\delta }}_{t-\Delta t}$$

The resulting effective traction force ensemble $\bar{T}$ is defined as follows:

(9) $$\bar{T}=\sqrt{\sigma_{n}^{2}+\sigma_{s}^{2}}$$

Since the current commercial finite element software ABAQUS (version 2021) only has a built-in CZM to address static load damage, other types of CZMs must be implemented by writing subroutines. Consequently, we utilized the user subroutine USDFLD to realize the aforementioned CZM, considering fatigue damage accumulation. 

**Figure 4:**
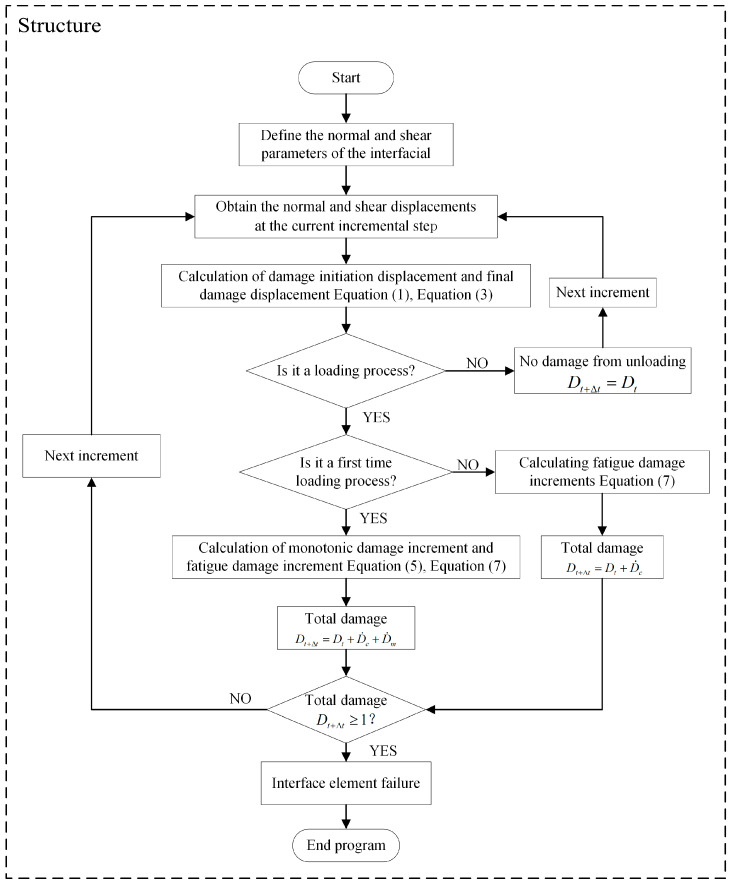
Flowchart of the implementation of the subroutine.

As illustrated in Figure 4, the flowchart outlines the overall procedure of the program. Building on this, the following section details the implementation of the method within the program. Firstly, the parameters of the cohesive element and the displacement at the current incremental step are obtained from the main program. Then, the damage initiation displacement and final damage displacement of the cohesive element under complex stress are calculated according to Equations (1) and (3). Once the displacement at the current incremental step exceeds the damage initiation displacement, the cohesive element will begin to undergo damage accumulation. Meanwhile, since this model integrates static damage and fatigue damage, the initial loading involves the calculation of static damage according to Equations (5) and (7). For subsequent loadings, only the accumulation of fatigue damage will be considered. If the element damage reaches 1 in the following incremental step, the element fails and is deleted.

### 2.2. Methods of Generating Aggregates at the Mesoscale

We generated a mesoscale model of concrete by random placement. At the same time, sand and cement were not characterized in detail in this study because the computational effort would be significantly increased if the shapes and sizes of all the mesoscale components were involved, so in this paper, the two were merged to simulate homogeneous mortar. The focus of the simulation study in this research is an RC beam with dimensions of 550 mm×150 mm×150 mm. Selecting the appropriate aggregate grading is crucial to achieving precise calculations within the mesoscale aggregate model. In this study, we utilized continuous grading, dividing the aggregate into intervals with specific proportions based on the aggregate size. This method effectively minimizes voids and enhances the overall compactness of the material, which is vital for structural integrity. Specifically, we generated aggregates within the 5–20 mm range by continuous grading. Moreover, aggregates with particle sizes smaller than 5 mm were classified as fine aggregates and incorporated into the mortar. The Fuller curve is widely recognized as the grading curve that gives the best working condition of concrete, and the percentage of each particle size component can be calculated based on the theory of maximum density curve proposed by Fuller [26] as follows:

(10) $$P(D_{0})=\sqrt{\frac{D_{0}}{D_{\max}}}$$

where $P\left(D_{0}\right)$ denotes the percentage of aggregate volume that passes through no more than the sieve diameter $D_{0}$; $D_{\max}$ denotes the maximum aggregate particle size.

Since Fuller’s grading formula calculates the actual optimum grading, which applies to three-dimensional problems, the research in this paper is based on a two-dimensional plane. It cannot directly use the above formula; it needs to use the Walraven formula [27] to convert Fuller’s grading to the grading curve of the particle size in a two-dimensional plane. The specific formula is as follows:

(11) $$P(D<D_{0})=P_{k}[1.065{\left(\frac{D_{0}}{D_{\max}}\right)}^{0.5}-0.053{\left(\frac{D_{0}}{D_{\max}}\right)}^{4}-0.012{\left(\frac{D_{0}}{D_{\max}}\right)}^{6}-0.0045{\left(\frac{D_{0}}{D_{\max}}\right)}^{8}+0.0025{\left(\frac{D_{0}}{D_{\max}}\right)}^{10}]$$

where $P(D<D_{0})$ denotes the percentage of aggregate area that passes through no more than the sieve diameter $D_{0}$. $P_{k}$ denotes the percentage of aggregate area. We take $P_{k}=0.6$ in this paper. According to Equation (11), the size of the distribution of the gradation sizes of all levels of the aggregate area with an area of 550 mm × 150 mm is calculated as shown in Table 1. The particle size of the rubber is controlled in the range of 1–3 mm.

After determining the proportion of aggregate at each range, it is essential to consider the impact of aggregate shape on the calculations. While various shapes like circular and elliptical aggregates are commonly referenced in existing research for their computational speed advantages [28,29,30], the aggregates used in the experiments are primarily crushed stone aggregates, so we simulated them by generating a random polygonal aggregate model through the program to be more consistent with reality.

**Figure 5:**
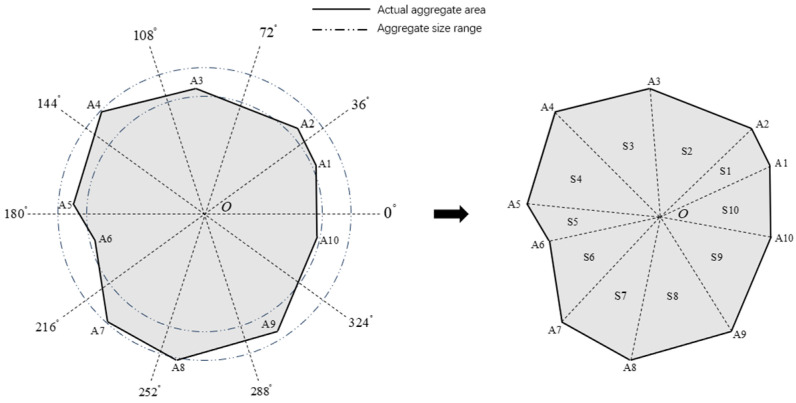
Polygonal aggregate generation.

We used the Python program to generate random polygonal aggregates by preprocessing ABAQUS. The vertex coordinates $\left(R,\theta \right)$ of the polygons are generated by a random function in the polar coordinate system, and the random function formula is as follows [31]:

(12) $$\left\{\begin{array}{l} R:R_{i}\times [1+Random(-1,1)\times f_{r}],\;\;0<f_{r}<1\;\\ \theta :\frac{2\pi }{\alpha }\times [i+Random(-1,1)\times f_{\theta }],\;0<f_{\theta }<0.5\; \end{array}\right.$$

where $R_{i}$ denotes the random radius of point $A_{i}$, the range of which is determined according to the aggregate grading range. *Random*( ) is a random function, indicating that a random number is generated from (−1,1). $f_{r}$ denotes the radius fluctuation coefficient with a range of 0–1, and the larger the value, the more pronounced the concavity and convexity of the aggregate’s edges. $f_{\theta }$ denotes the angular fluctuation coefficient with a range of 0–0.5, and the larger the value, the more pronounced the angle of deflection. α denotes the number of individual aggregate edges. i denotes the number of aggregate corner points. By inputting the parameters, a single aggregate can be generated, as shown in Figure 5. When the number of aggregate corner points is greater than 2, the program will automatically calculate the triangle area $S_{1},S_{2}\cdots S_{i}$ and sum up to obtain the area of a single aggregate.

Utilizing Formula (12), the random generation of Table 1 is repeated within the particle size range of a single aggregate, and the total area of all aggregates is calculated until the total area of the aggregate fulfills the requirements of the current gradation ratio. Subsequently, the model proceeds to the next level of matching to continue generating aggregates randomly. Eventually, the generation of graded aggregates at all levels will be completed, and through the Python script, the graded aggregate information will be stored at all levels. This information is imported into ABAQUS for parametric modeling.

### 2.3. Methods of Generating Cohesive Elements

After constructing the stochastic aggregate model of RC beams, it is essential to consider the modeling of the ITZ. It is situated at the junction of different materials and is characterized by low strength and fracture energy [32]. In this study, zero-thickness cohesive elements were employed to model the ITZ. Although conventional approaches also utilize solid elements with actual thickness, the extremely thin nature of the ITZ layer complicates the modeling process. Furthermore, while increasing the thickness of the ITZ is permissible, it results in excessive mesh density and computational challenges. Therefore, we adopted zero-thickness cohesive elements to characterize the ITZ.

**Figure 6:**
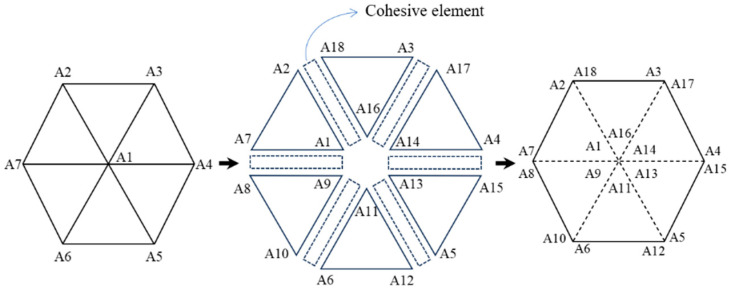
Insertion of zero-thickness cohesive elements.

**Figure 7:**
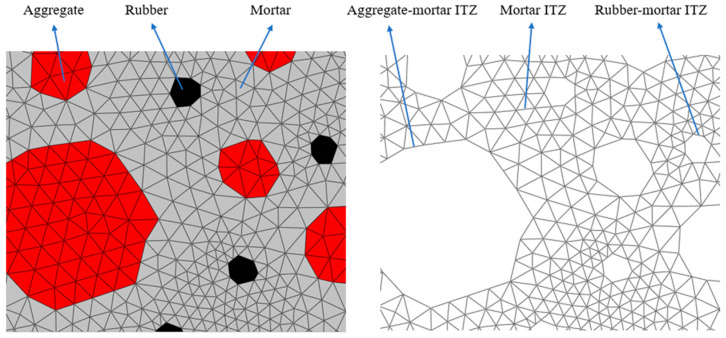
Aggregate and cohesive elements generated in ABAQUS.

In this study, the batch insertion of cohesive elements was achieved by writing Python scripts. Firstly, the script reads the element node numbers in the model’s input file and then finds the common nodes of the pre-inserted cohesive zone elements. Subsequently, following the flowchart presented in Figure 6, the insertion of cohesive zone elements can be accomplished by modifying the common node numbers of the elements in the input file in a batch. Finally, the cohesive elements of each phase of the RC solid element and the transition zone of each phase interface are generated, as shown in Figure 7.

### 2.4. Model Parameters

We considered aggregate, mortar, and rubber particles as linear, elastic solid elements. Zero-thickness cohesive elements were inserted between the aggregate–mortar interface, the rubber–mortar interface, and the internal element interface of the mortar, which were in turn used to simulate the crack extension of RC beams under fatigue loading. Xiong et al. [33] and others implemented numerical simulations of the tension and compression of concrete using a cohesive model. This study builds upon their work to establish the material parameters for the aggregate mortar and the ITZ between them. The parameters of the rubber, on the other hand, were determined from uniaxial tensile experiments on RC according to the literature [34]. The material parameters are outlined in Table 2 and Table 3.

## 3. Model Verification

### 3.1. Experimental Data

To verify the model’s feasibility, we compared the simulated results with experimental data on RC beams’ peak under static loads and their fatigue life under fatigue loads.

The comparative experiment data referenced in this paper were sourced from the studies of Liu et.al [35]. Liu designed experiments to assess the fatigue performance of RC beams subjected to unidirectional loading. These included static load and fatigue loading experiments. The specimens used were standard-sized RC beams measuring 150 mm×150 mm×550 mm. First, Liu conducted static loading experiments on RC beams with varying rubber replacement rates using a digital tester to obtain the load–deflection curves and record the peak loads. Subsequently, a three-point bending fatigue experiment was performed on the RC beams with a span of 400 mm using a 500 kN electrohydraulic servo tester to determine the fatigue life of the beams. Finally, the fatigue life curve for the RC beams was constructed. During this process, Liu controlled the applied load to be a uniform pulsating load of equal amplitude, which was set at a discounted value of the peak load, with a load ratio S maintained at 0.1 ($S=P_{min}/P_{max}$).

The cement utilized in [35] was ordinary silicate cement with a compressive strength of 42.5 MPa (maintained for 28 days). Natural river sand, with a fineness modulus of 2.40, served as the fine aggregate, while continuously graded crushed stone aggregate was used as the coarse aggregate for the specimens. Additionally, rubber particles measuring 2 mm in size replaced equal volumes of fine aggregates at replacement rates of 0%, 5%, 10%, and 15%. The specific mix ratios employed in the experiments are detailed in Table 4.

### 3.2. Mesoscale Modeling of Rubber Concrete

**Figure 8:**
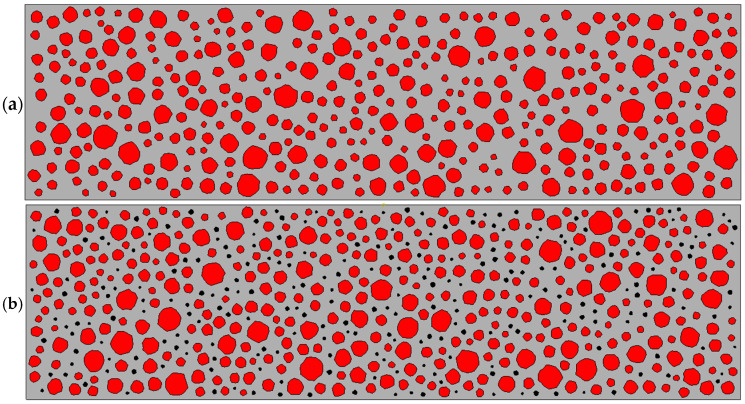

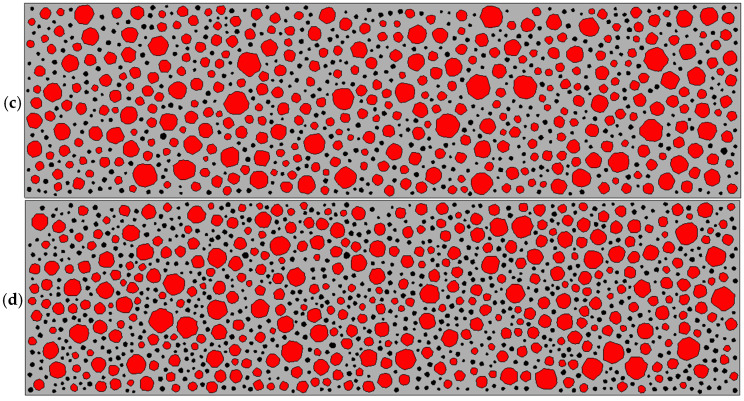
Mesoscale modeling of concrete beams with various rubber replacement rates: (**a**) 0%; (**b**) 5%; (**c**) 10%; (**d**) 15%.

Based on Table 1, the area of continuously graded aggregates was calculated using Walraven’s formula. The mortar was replaced with rubber particles at 0%, 5%, 10%, and 15% to produce RC beams with varying rubber replacement rates, as illustrated in Figure 8. In the figure, red denotes aggregate particles, black represents rubber particles, and gray signifies mortar.

**Figure 9:**
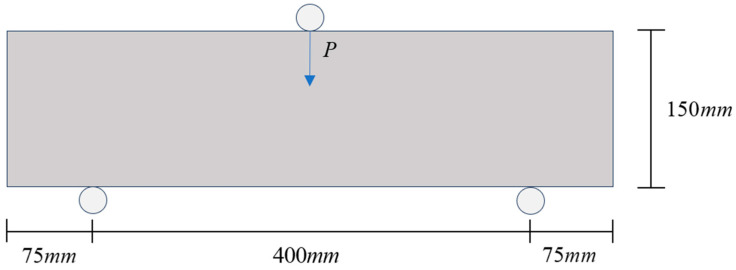
Schematic of three-point bending loading.

After establishing the aggregate model, the load was applied at the midpoint of the top of the beam following the loading configuration of the experimental three-point bending setup. The distance between the two supports at the bottom was 400 mm, and the distance from the supports to the left and right boundaries was 75 mm, as depicted schematically in Figure 9.

**Figure 10:**
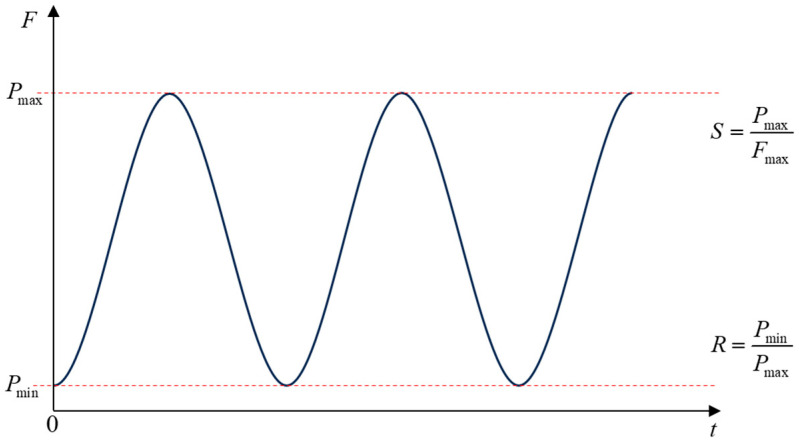
Load amplitude function graph.

We used the finite element software ABAQUS (version 2021, Dassault Systèmes, France) to solve the model. Initially, a simulation of three-point bending under static load was conducted on the beam. Displacement load was applied at the top of the beam to derive the load–deflection curve, with the maximum load value denoted as the peak load of the model, $F_{max}$. Subsequently, fatigue loading simulations were performed using various stress levels corresponding to the member’s peak load. The stress ratio levels S were set to 0.9 and 0.8 based on comparative experiments (where $S=P_{max}/F_{max}$, with $P_{max}$ being the maximum fatigue load during loading). Fatigue loading followed a sinusoidal cycle amplitude function controlled method based on experimental conditions. The computational analysis involved loading–unloading steps of 1 time unit, with a stress ratio R set to 0.1 ($R=P_{min}/P_{max}$, where $P_{min}$ represents the minimum fatigue load during loading). The amplitude function curve is illustrated in Figure 10.

### 3.3. Comparative Results

In this subsection, the simulation results of three-point bending static load and fatigue loading of RC beams are compared with the corresponding experimental results.

#### 3.3.1. Three-Point Bending Static Load Simulation

**Figure 11:**
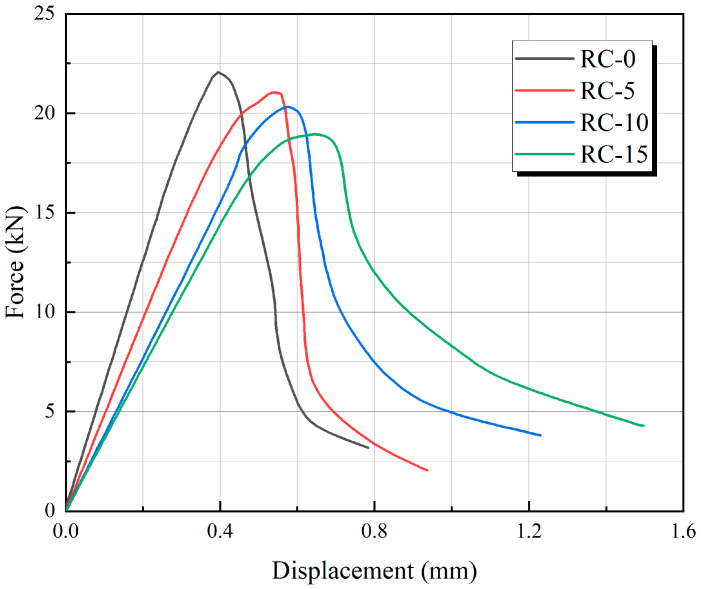
Load–deflection curve.

Figure 11 shows the load–deflection curves from the RC beam’s three-point bending static load simulation. The figure shows that the load–deflection curve of ordinary concrete has steeper rising and falling segments, which is apparent brittle damage. In contrast, the load–deflection curves of RC show a gentle upward and downward trend with an increase in peak deflection, and this trend is more pronounced with the rise in the rubber replacement rate. This shows that adding rubber particles to concrete members can improve the toughness of concrete, which is consistent with the conclusions of the experiments.

**Figure 12:**
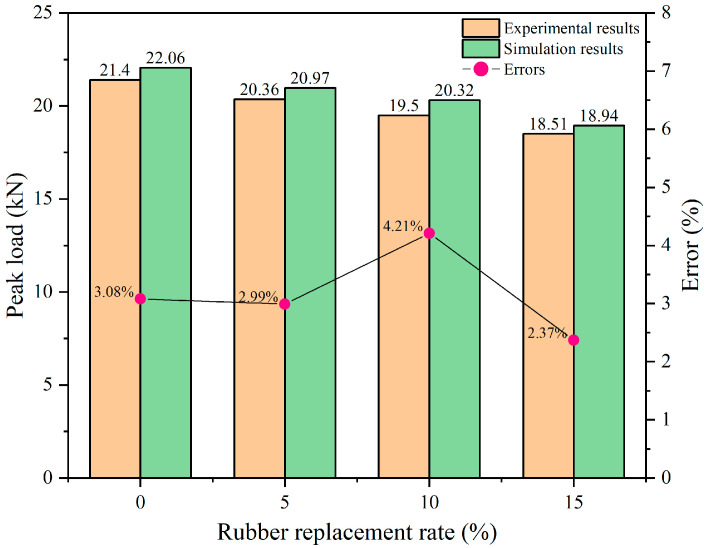
Comparison of experimental and simulated peak load errors.

In addition, the peak load of each curve in Figure 11 is extracted and compared with the peak load obtained from experiments. The error values between the simulation and experiment are analyzed and presented in Figure 12. The comparison shows that the error between simulation and experimental results is relatively small, with a maximum absolute error of 4.2%. As the rubber replacement rate increases, the peak load in the figures exhibits a decreasing trend, consistent with experimental findings. This further validates the model’s reliability in predicting the beam’s peak three-point bending static load.

#### 3.3.2. Three-Point Bending Fatigue Loading

We simulated the three-point bending fatigue loading of concrete beams with different rubber replacement rates, and the fatigue life at various stress levels was derived. The simulated fatigue life was then compared with experimentally obtained to assess the model’s reliability in predicting fatigue behavior.

**Figure 13:**
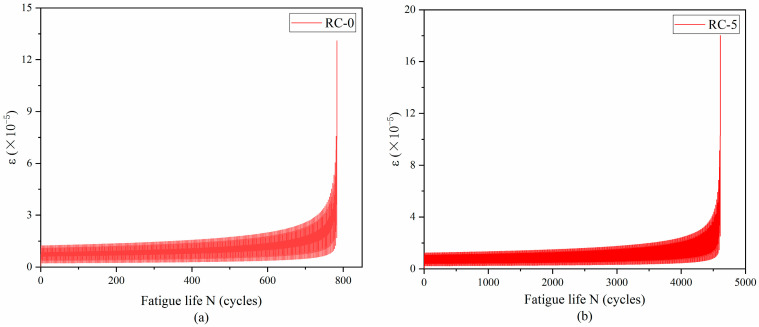

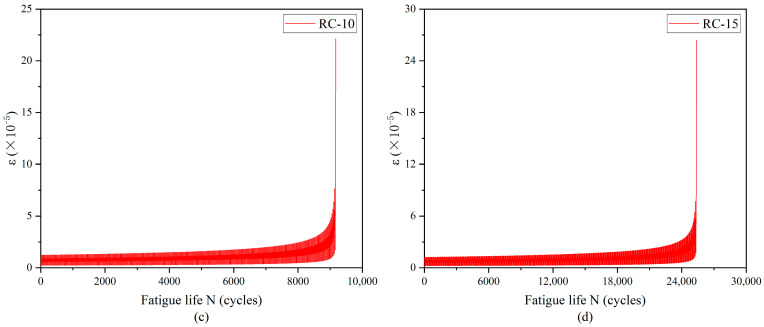
S = 0.9 Simulated strain–cycle number curves for three-point bending fatigue loading. Rubber replacement rate: (**a**) 0%, (**b**) 5%, (**c**) 10%, (**d**) 15%.

**Figure 14:**
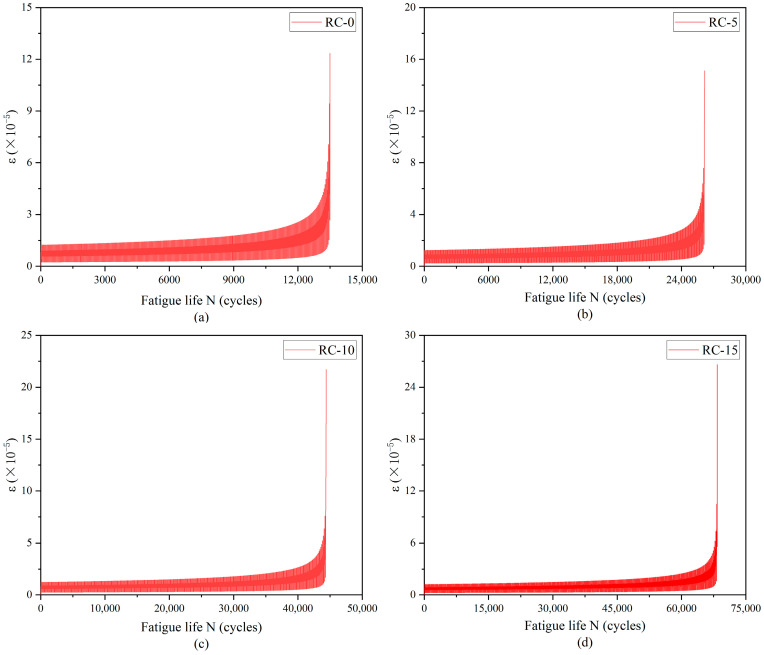
S = 0.8 Simulated strain–cycle number curves for three-point bending fatigue loading. Rubber replacement rate: (**a**) 0%, (**b**) 5%, (**c**) 10%, (**d**) 15%.

The transverse strain at the bottom of the beam span was plotted against the number of loading cycles depicted in Figure 13 and Figure 14. The figures illustrate a gradual increase in transverse strain with the number of cycles, culminating in a sharp change indicative of macroscopic crack formation at beam failure. This pattern aligns closely with the strain-cycle number curve observed in experimental results.

Table 5 summarizes the fatigue life results from both experiments and simulations. The fatigue life of RC beams varies in the experiments, even when made with the same mix ratio, due to differences in internal aggregate distribution. However, from an overall point of view, the fatigue life is increased with the increase in the rubber replacement rate. Therefore, in this paper, when verifying the reliability of the fatigue life calculated by the model, the maximum and minimum values of the fatigue life obtained from the experiments were used as the basis. If the simulation results are within this range, it proves that the fatigue life calculated by the model is reliable.

**Figure 15:**
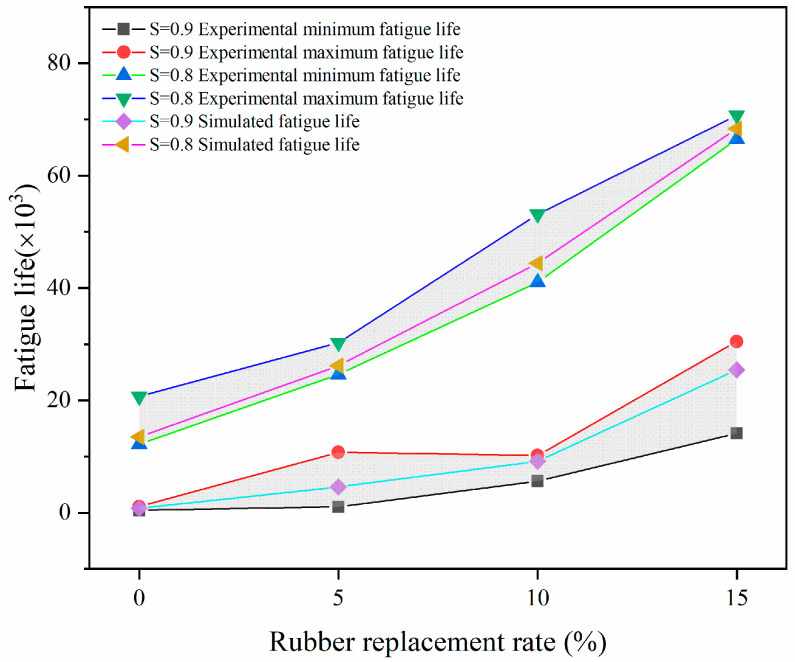
Experimental and simulated fatigue life: shaded gray area represents the range between maximum and minimum values.

As shown in Figure 15, the fatigue life results derived from the simulations consistently fall within the range defined by the minimum and maximum values of the experimentally determined fatigue life. This reinforces the model’s reliability in predicting fatigue life.

## 4. Results

In this study, we conducted a three-point bending fatigue loading simulation on RC with rubber replacement rates of 0%, 5%, 10%, and 15%. By adding S = 0.75 and S = 0.85 to the existing S = 0.8 and S = 0.9, we derived the fatigue life, the fatigue damage form of the RC, and the damage evolution law along the cross-section.

### 4.1. Damage Forms of Rubber Concrete under Fatigue Loading

**Figure 16:**
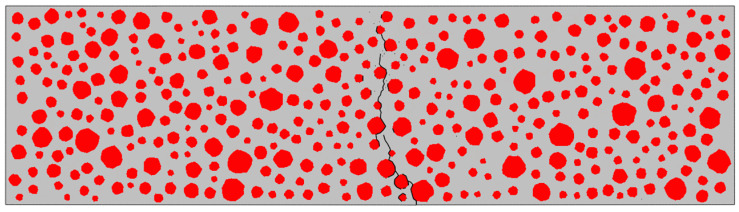
Forms of fatigue damage in ordinary concrete volumes: cracks represented by the black line.

In this paper, the fatigue damage of RC is manifested through the failure deletion of cohesive elements. At higher stress levels, the damage to the cohesive elements gradually accumulates with each loading–unloading cycle, decreasing the interface strength until it can no longer carry the load, eventually leading to cracking. As shown in Figure 16, the damage form of an ordinary concrete beam with dimensions of 550 mm×150 mm was simulated by three-point bending fatigue loading at S = 0.9. Since the damage of the beam under three-point bending loading occurred in the middle of the span, a sight window of 150 mm×150 mm in the middle of the beam was taken for observation for convenience.

**Figure 17:**
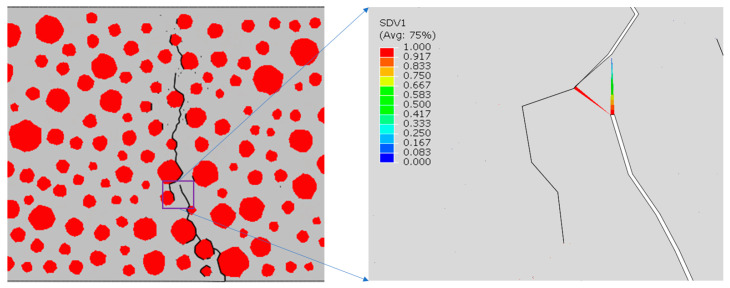
Re-expansion of fatigue cracks.

The re-expansion of the crack’s path seeks the path of least resistance. As observed in the magnified cloud image of the crack in Figure 17, the cohesive elements in the mortar exhibit low damage levels, allowing for further accumulation of fatigue damage (SDV1 was used in this study as a subroutine state variable to quantify fatigue damage accumulation). However, the cohesive elements in the bone–mortar transition zone on the left have already failed and been removed due to fatigue loading. Consequently, the cracks predominantly re-expand along the left interface of the aggregate–mortar ITZ.

**Figure 18:**
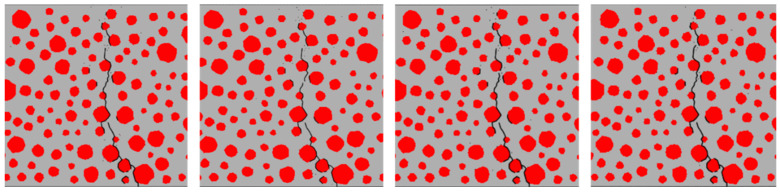
Forms of fatigue damage in ordinary concrete beams.

**Figure 19:**
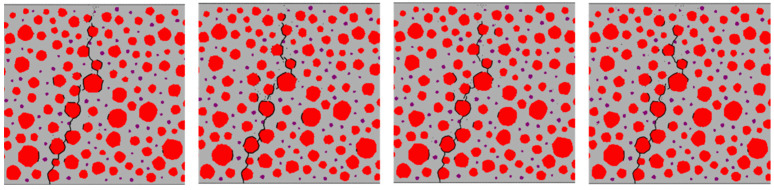
Forms of fatigue damage in 5% rubber replacement rate concrete beams.

**Figure 20:**
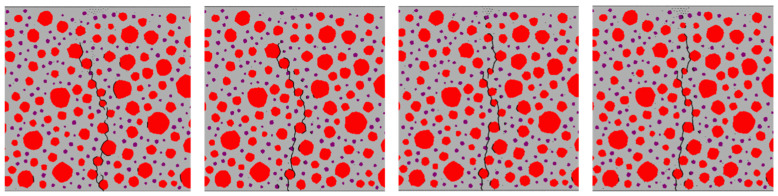
Forms of fatigue damage in 10% rubber replacement rate concrete beams.

**Figure 21:**
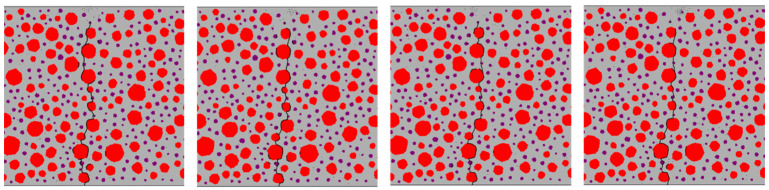
Forms of fatigue damage in 15% rubber replacement rate concrete beams.

Figure 18, Figure 19, Figure 20 and Figure 21 show the concrete beams’ fatigue loading damage forms with 0%, 5%, 10%, and 15% rubber replacement rates at four stress levels, respectively.

Figure 18 compares the fatigue loading damage forms of the same concrete beam with a 0% rubber replacement rate at four stress levels. The figure shows that when the concrete beam without rubber is subjected to fatigue loading, there is no change in the fatigue damage cracks at the aggregate interface under different stress levels. The overall expansion path of the cracks tends to be consistent, extending upward from the same point at the bottom of the beam along the aggregate interface near the bottom and branching out more in the path of crack expansion. This indicates that the internal force distribution in ordinary concrete under fatigue loading is relatively uniform, and the crack extension’s minimum resistance path remains unchanged.

In Figure 19, fatigue damage cracks at the transition zones of each aggregate–mortar interface in concrete with a 5% rubber replacement rate exhibit significant variation across different stress levels. Larger aggregate particles correlate with more severe damage cracks. While subtle branching appears at intermediate aggregate interfaces, the overall trend remains consistent.

Figure 20 illustrates substantial differences in crack extension paths between stress levels of 0.9 and 0.85 versus 0.8 and 0.75 for concrete with a 10% rubber replacement rate. Higher stress levels show cracks extending along a single path, whereas lower stress levels exhibit significant branching, particularly around densely placed rubber particles.

Figure 21 shows that at a 15% rubber replacement rate, crack extension paths are confined to areas densely populated with rubber, with minimal branching in the mortar across varying stress levels. Notably, at a stress level of 0.9, fatigue crack extension paths in RC differ significantly from those observed at other stress levels, where paths at 0.85, 0.8, and 0.75 stress levels demonstrate more stable patterns.

### 4.2. Fatigue Life of RC

Table 6 summarizes the fatigue life of simulated RC at different stress levels. The results indicate that the fatigue life of RC increases as the stress level decreases, given a constant rubber substitution. When the stress level decreased from 0.9 to 0.75, the most significant change in fatigue life was observed for a 15% rubber substitution, and the slightest change occurred for a 0% rubber substitution. Additionally, the fatigue life of RC improved with an increasing rubber rate at the same stress levels. Specifically, at these four stress levels, the fatigue life of RC with a 10% rubber substitution was 26, 7, 4.6, and 3.7 times greater than that of RC with a 0% rubber substitution, respectively.

**Figure 22:**
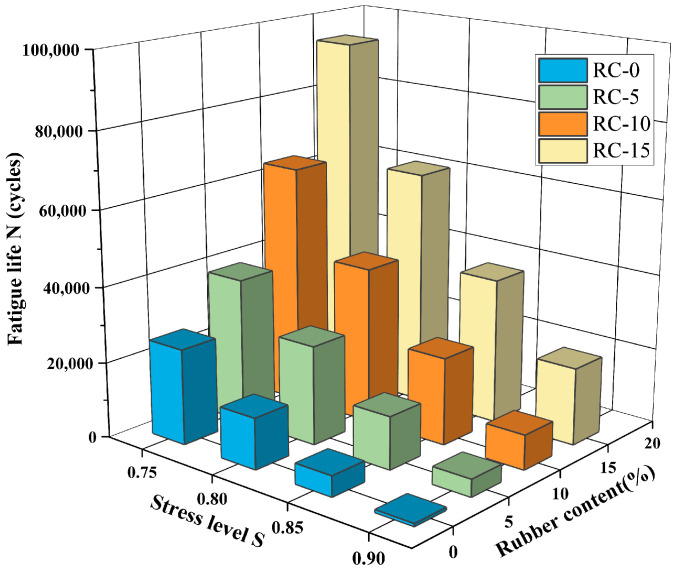
Fatigue life histogram.

**Figure 23:**
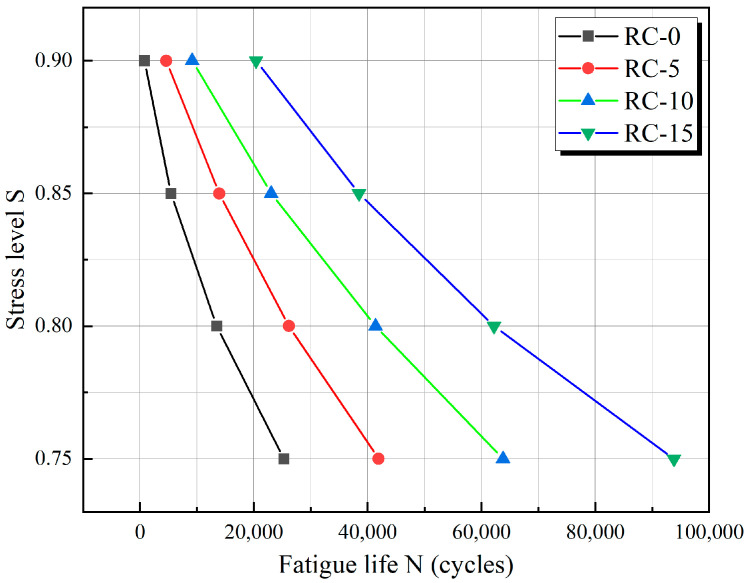
Fatigue life relationship diagram.

The histogram shown in Figure 22 clearly shows the association between the rubber replacement rate, fatigue life, and stress level S. The figure shows that RC’s fatigue life is related to the rubber replacement rate and stress level. The fatigue life increased significantly within a certain range when the rubber replacement rate increased, and the stress level was low. In contrast, the fatigue life decreased when the rubber replacement rate was low, and the stress level was high. Figure 23 shows the trend of a negative correlation between fatigue life and stress level for the same rubber replacement rate.

### 4.3. Interfacial Damage Evolution Laws

**Figure 24:**
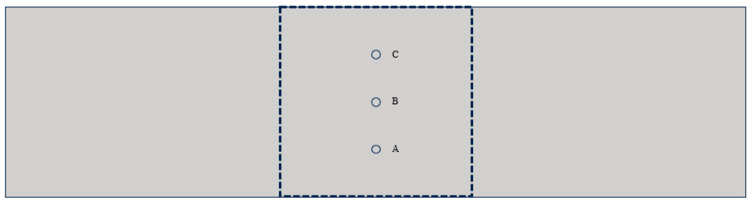
Schematic diagram of the pickup point: Point A (Bottom), Point B (Middle), and Point C (Top).

In order to investigate the fatigue damage evolution law at different locations of the beam cross-section, we took points at equal spacing along the mid-span cross-section of the beam from the bottom to the top, as shown in Figure 24.

**Figure 25:**
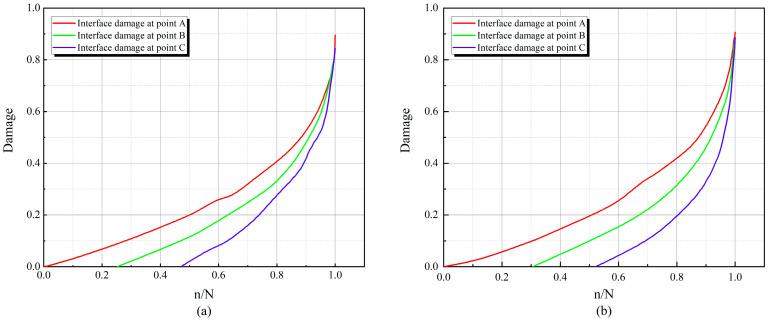
Interfacial damage evolution curves for RC-0: (**a**) S = 0.9, (**b**) S = 0.8.

**Figure 26:**
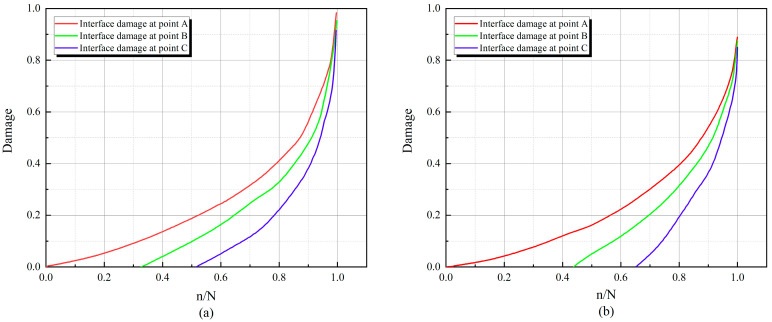
Interfacial damage evolution curves for RC-10: (**a**) S = 0.9, (**b**) S = 0.8.

The extent of mortar damage near the crack at this point was extracted during the fatigue loading process. Evolution curves are shown in Figure 25 and Figure 26. N indicates fatigue life, and n/N indicates relative fatigue life. From the figure, it can be observed that the damage evolution of the interface proceeds upward sequentially along the mid-span cross-section of the beam during the whole fatigue loading process. The damage accumulation rate of the interface slows down as the stress level decreases. Meanwhile, compared with ordinary concrete, the damage initiation at the interface of RC is lagging more, and the damage accumulation is slower.

## 5. Discussion

### 5.1. Analysis of Fatigue Damage Causes of RC

The fatigue crack extension forms were obtained by simulating the fatigue loading of RC beams with different rubber admixtures (Figure 18, Figure 19, Figure 20 and Figure 21). The simulation results are consistent with the fatigue damage form of the three-point bending fatigue experiments on RC in the literature [35,36]. They all started cracking from the bottom and formed a significant crack extending to the middle and upper part of the beam. According to Figure 17, it can be seen that the weak nature of ITZ makes it easier for the cracks to re-expand along the boundaries of the aggregate. In particular, the boundaries of larger aggregate particles are more susceptible to cracking due to stress concentration. Rubber particles have good elasticity and act like tiny springs in concrete, being able to dissipate the energy generated by external loading through deformation until the interface fails. The fatigue cracks in the concrete with a 5% rubber replacement rate expanded along the boundaries of large aggregate particles and rubber particles with fine branching in the mortar due to the low rubber content. At a rubber replacement rate of 10%, crack branching in the mortar was further reduced because the rubber and the aggregate–mortar interface shared more of the fatigue loading energy. At a rubber replacement rate of 15%, the increased rubber content and its dissipated energy further suppressed fatigue cracks in the mortar, resulting in cracks expanding along a single main crack at the phase interfaces. Compared to the concrete fatigue model developed based on plasticity theory [37,38], the model proposed in this study can simulate crack propagation better. This improvement is attributed to directly integrating CZM elements into the RC as zero-thickness elements. Crack expansion can occur spontaneously as fatigue loading progresses according to the damage. In contrast, the plasticity theory model is reflected in the solid elements in the form of damage dispersion, which does not allow for visual observation of crack expansion. Thus, the CZM model demonstrates superior capability in simulating crack propagation compared to the fatigue model based on plasticity theory.

In practical experiments, it is impossible to load RC beams with the same aggregate distribution at different stress levels [35,36]. This is because the aggregate distribution within the beam is randomized during fabrication, leading to varying peak three-point bending static loads for each beam. Although the same stress level can be applied, the aggregate distribution affects the final fatigue life results. Using simulation can lead to this, and this is where mesoscale simulation has a unique advantage.

### 5.2. Fatigue Life Analysis of Rubber Concrete

The most important index for judging the fatigue performance of RC is fatigue life under certain conditions. The fatigue life of RC beams with varying rubber replacement rates was calculated using the model proposed in this study and compared with experimental results (see Figure 15). The findings indicate that the calculated fatigue life falls within the range of the experimentally obtained values, demonstrating the model’s validity for estimating fatigue life.

Considering that the stress level–fatigue life curve of Figure 23 has a simple nonlinear form, it has limitations in providing a basis for practical experiments. Therefore, we further analyzed the correlation between stress level and fatigue life based on the fatigue life obtained from the simulation above. Empirically, the stress level S is associated with the natural logarithm of the fatigue life [39]. Based on the simulation results and by testing different fitting equations, we assumed that the following equation can describe the nonlinear relationship between stress level and fatigue life:

(13) $$S=\alpha +\beta \ln(N)+\gamma \ln^{2}(N)\;$$

where *S* denotes the stress level of RC, and *N* denotes the fatigue life of RC. α, β, and γ denote factors related to admixtures.

The simulation results shown in Table 6 were fitted using Equation (13), and the resulting fatigue life equations, along with the coefficient of determination $R^{2}$ for each RC replacement rate, are presented in Table 7. It can be seen that the fitted equation satisfies the correlation requirement. This also shows that the stress level is associated with a logarithmic function of the fatigue life through this equation.

**Figure 27:**
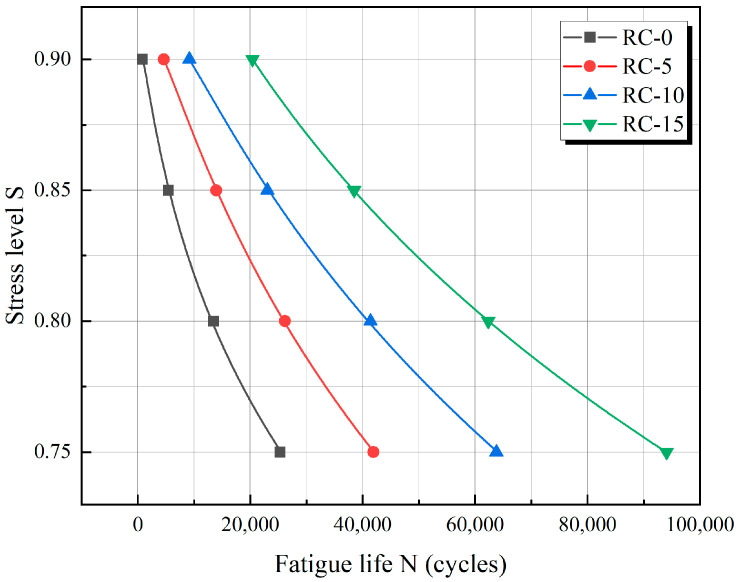
Fitted curves of stress levels and fatigue life of RC with different rubber replacement rates.

The resulting curve is plotted in Figure 27. The nonlinear relationship between S and N under different rubber replacement rates is established based on the fitting results. Liu [35] conducted fatigue experiments on RC at medium and high stress levels, utilizing a double logarithmic equation to construct the fatigue life equation while analyzing fatigue performance. However, recent research [40] indicates that the double logarithmic fatigue equation is more appropriate for analyzing fatigue at low and medium stress levels. In contrast, the fatigue equation proposed in this paper introduces a second-order term, making it better suited for a more complex nonlinear description of fatigue behavior at medium and high stress levels.

### 5.3. Interface Damage Evolution Analysis

As shown in Figure 25 and Figure 26, during the fatigue loading process, the damage evolution of the interface follows a specific pattern. The interface near the bottom of the beam produces damage first, while damage at the interface in the upper middle part tends to lag, consistent with findings from previous studies [41]. This phenomenon is because the model presented in this paper distinguishes between the tensile and compressive behavior of the cohesive element. At the beginning of loading, the bottom of the RC beam is subjected to tension, and the top is subjected to compression. At the same time, the compressive strength of RC is much larger than the tensile strength of the beam (this has been confirmed by the fatigue experiments conducted by Walid [42]), which leads to the fact that the interface element at the bottom of the beam is more susceptible to damage due to the tension. As fatigue loading proceeds, the tensile region extends upward, leading to a change in the stress state of the interface elements and a lag in the accumulation of damage in the middle and upper portions of the interface elements.

By comparing (a) and (b) in Figure 25 and Figure 26, respectively, it can be found that at the same replacement rate, the curves under higher stress levels are steeper, while the curves under lower stress are relatively flat. This indicates that the damage accumulation rate at the interface will be faster at higher stress levels. This leads to the fact that the RC beams at higher stress levels at the same replacement rate will be damaged first and have a shorter fatigue life. By comparing (a) and (b) in Figure 25 with (a) and (b) in Figure 26, it is found that the interfacial damage accumulation of RC is lagging more at the same stress level, which results in the RC being able to withstand more fatigue loadings.

### 5.4. Potential Applications and Perspectives

The CZM developed in this paper, which incorporates fatigue damage accumulation, represents a new approach to numerical calculation. The model can accurately simulate the fatigue crack extension of RC beams under three-point bending fatigue loading and derive the fatigue life of the beams, as well as understand the fatigue performance of RC under different loading stress levels.

Furthermore, the research object of this model is RC; for ordinary concrete or concrete containing other mortar replacements, the difference lies in the internal mortar replacements and the internal component differences. By modifying the material properties and generating a specific internal component relationship, the model can be utilized for fatigue life prediction research. The model proposed in this paper is also suitable for fatigue loading simulations that require modeling interfacial layers with thickness. For example, the interfacial layer between the old and new mortar of recycled concrete aggregates can also be characterized using the model proposed in this paper.

In addition, the model proposed in this study is currently applicable only to calculating two-dimensional models. The force conditions in three-dimensional models are more complex. To enhance the generality of this model, incorporating the second shear stress into the CZM is recommended for subsequent studies, where its feasibility can also be further verified.

## 6. Conclusions

This paper proposes a CZM that accounts for the accumulation of fatigue damage at the mesoscale to investigate the fatigue performance of RC. The model integrates static and fatigue damage within the CZM and can effectively capture the damage to RC resulting from fatigue loading. After verifying the model’s accuracy, numerical simulations were conducted on concrete beams with varying rubber replacement rates, and their fatigue performance was analyzed. Based on the simulations, we draw the following key conclusions:

(1) Under fatigue loading, the fatigue crack extension of RC with a 0% rubber substitution rate is simple and extends along the aggregate boundary. The fatigue crack extension of RC with a 5% rubber substitution rate mainly extends along the boundary of aggregate and rubber. The crack branching of RC with a 10% rubber substitution rate in the mortar is reduced. In comparison, the crack of RC with a 15% rubber substitution rate forms a single main crack along the boundary of aggregate and the boundary of rubber. The primary reason for these phenomena is that the incorporation of rubber inhibits the expansion of microcracks.
(2) As the rubber substitution rate increases, the fatigue life of RC exhibits an upward trend at the same stress level. This improvement is attributed to the favorable deformation properties of the rubber particles, which dissipate energy from external loading, allowing the RC to withstand more fatigue loads. Additionally, based on the fatigue life of RC obtained from the simulations, a fatigue equation that is more suitable for medium and high stress levels is proposed.
(3) At the same rubber replacement rate, the damage accumulation rate of the RC interface at high stress levels is faster. At the same stress level, the time of interface damage of RC with a 10% rubber replacement rate lags behind that of RC with a 0% rubber replacement rate. This phenomenon occurs because a higher stress level causes the tensile and compressive state of the interface to change faster. Rubber particles delay the fatigue damage accumulation rate of the interface element at the bottom of the beam, resulting in a lag in transforming the tensile and compressive state of the upper interface element in the beam span.

The model developed in this study effectively simulates and analyzes the fatigue performance of RC and can be applied to any type of RC through improvements to the fine-scale model, providing an important reference for using RC in construction, roads, bridges, and other engineering fields.

## Figures and Tables

**Table 1:** Total area of aggregate generation at all levels.

Rubber Content (%)	5–10 mm (mm^2^)	5–10 mm (mm^2^)	5–10 mm (mm^2^)	Rubber (mm^2^)
0	10,754	7587	4464	0
5	10,754	7587	4464	1335
10	10,754	7587	4464	2669
15	10,754	7587	4464	4004

**Table 2:** Material parameters of solid elements.

Parameter	Aggregate	Mortar	Rubber
Modulus of elasticity (GPa)	72	36	7
Poisson’s ratio	0.16	0.2	0.495

Note: Aggregate and mortar parameters are from [33], and rubber parameters are from [34].

**Table 3:** Parameters of cohesive elements.

Parameter	Mortar ITZ	Aggregate–Mortar ITZ	Rubber–Mortar ITZ
Normal strength (MPa)	4	2.6	1.82
Shear strength (MPa)	30	10	7
Normal fracture energy (N/mm)	0.1	0.025	0.0175
Shear fracture energy (N/mm)	2.5	0.625	0.438

Note: Aggregate and mortar ITZ parameters are from [33], and rubber ITZ parameters are trial values.

**Table 4:** Experimental mixing ratios.

Type	Material (kg/m^3^)					
	Water	Cement	Sand	Gravel	Rubber	Water Reducer
RC-0	131.5	420	555	1296	0	5.0
RC-5	131.5	420	527	1296	11.68	5.0
RC-10	131.5	420	500	1296	22.95	5.0
RC-15	131.5	420	472	1296	34.66	5.0

**Table 5:** Comparison of experimental and simulation results.

		0%	5%	10%	15%
S = 0.9	Experimental Min/Max	450/1090	1070/10,745	5653/10,176	14,134/30,465
	Simulation	783	4607	9175	25,395
S = 0.8	Experimental Min/Max	12,215/20,686	24,587/30,250	40,997/53,110	66,498/70,716
	Simulation	13,496	26,171	44,398	68,346

**Table 6:** Fatigue life of RC.

	RC-0	RC-5	RC-10	RC-15
S = 0.9	783	4607	9175	20,395
S = 0.85	5450	13,936	23,058	38,462
S = 0.8	13,496	26,171	41,398	62,194
S = 0.75	25,279	41,921	63,793	93,807

**Table 7:** Fitting formulas.

Rubber Replacement Rate	Fitting Function	R^2^
0%	$S=0.406+0.151\ln(N)-0.012\ln^{2}(N)$	0.995
5%	$S=-0.408+0.332\ln(N)-0.021\ln^{2}(N)$	0.998
10%	$S=-0.751+0.394\ln(N)-0.023\ln^{2}(N)$	0.993
15%	$S=-0.556+0.359\ln(N)-0.021\ln^{2}(N)$	0.998

## Data Availability

The data used in the article can be obtained from the author here.
